# *Moreiba* gen. n., a new Canarian genus in Laparocerini (Coleoptera, Curculionidae)

**DOI:** 10.3897/zookeys.333.6122

**Published:** 2013-09-20

**Authors:** Miguel A. Alonso-Zarazaga

**Affiliations:** 1 Depto. de Biodiversidad y Biología Evolutiva, Museo Nacional de Ciencias Naturales (CSIC), José Gutiérrez Abascal, 2, E-28006 Madrid, Spain

**Keywords:** Weevils, Laparocerini, *Moreiba*, *Strophosoma canariense*, new genus, new combination, morphology, systematics, Canary Islands

## Abstract

A new genus *Moreiba* is described for *Strophosoma canariense* Franz, 1995 (type species) and placed in Laparocerini. It differs from *Laparocerus* Schoenherr, 1834 by the small size, the strongly transverse rostrum, the dense longitudinal strigosity on head and rostrum, the body covered by dense, adpressed scales and short, semierect subspatulate to parallel setae, the slender antennae with bisinuate scape and short oval club, the granulate pronotum and all tibiae lacking a mucro in both sexes. *Moreiba canariensis* (Franz, 1995), **comb. n.**, is the only described species, distributed in El Hierro and Gran Canaria. The tribal placement of the genera *Aphyonotus* Faust, 1895, *Asmaratrox* Heller, 1909, *Straticus* Pascoe, 1886 and *Cyrtozemia* Pascoe, 1872 is discussed.

## Introduction

Herbert Franz (1995) described a new species of Canarian weevil as *Strophosoma canariense* (type locality: El Hierro, Las Playas) based on a holotype and 81 paratypes, coming from three localities on El Hierro (Las Playas, La Dehesa, El Sabinal) and one in Gran Canaria (Isleta). Reading the description, I noticed I had some similar specimens from Lanzarote sent by Gunnar Israelson and Lothar Dieckmann several years before for identification. I requested some specimens from Herbert Franz for study and he kindly sent me a dozen as a present for my collection. My materials and those from Franz belong to two different species of the same genus. Later some colleagues have found more populations in different islands, so I have decided to describe the genus as new (the original placement in *Strophosoma* Billberg, 1820 is incorrect), to allow them to publish their own discoveries under a legitimate generic name.

## Material and methods

The specimens were studied under a binocular Leica Wild MZ8 microscope and photographed with an Olympus C7070WZ camera mounted on the same microscope. Microscope slides were studied and photographed with the same camera mounted on a Leitz Diaplan microscope, and some details were drawn by using a drawing tube. Extended focus images were generated using Alan Hadley’s software CombineZP. The programs Adobe Illustrator CS5.0 and Adobe Photoshop CS5.0 were used for image postproduction and mounting. The description follows the usual terminology in Curculionidae, especially that in use in Machado (2010). Dissection methods follow Alonso-Zarazaga (1990). Genitalia and terminalia have been placed in a drop of DMHF on an acetate card accompanying the specimen for long-term conservation (Steedman 1958; Bameul 1990). Body length is measured from the midpoint of front margin of pronotum to the most apical point of the elytra (the apex is hidden under the overhanging declivity) in dorsal view, width is measured at the widest point of the elytra in dorsal view. In other structures, length and width are measured at the maximum points, unless otherwise stated.

## Taxonomy

### Moreiba

Alonso-Zarazaga gen. n.

http://zoobank.org/2619B147-58F9-478F-9318-B567197CB90E

http://species-id.net/wiki/Moreiba

Figs 1 – 14

#### Type species.

*Strophosoma canariense* Franz, 1995, by present designation.

#### Diagnosis.

Small apterous Laparocerini with very short and wide rostrum; head and rostrum with a dense longitudinal strigosity; body covered by dense, adpressed scales; antennae distinctly slender with bisinuate scape and short oval club; pronotum granulate; elytra elliptical, weakly convex with declivity overhanging apex; legs with all femora edentate and all tibiae lacking mucro in both sexes, and endophallus devoid of visible sclerites.

#### Description.

**Figures 1–4.**
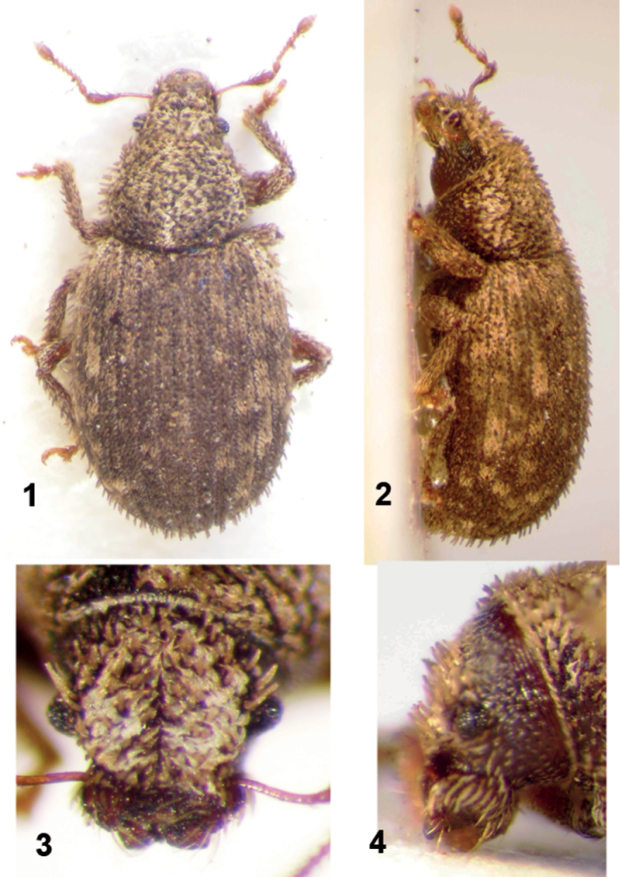
*Moreiba canariensis* (Franz): **1** Habitus, dorsal **2** Habitus, lateral **3** Head, dorsal **4** Head, lateral.

*Body* (Figs 1–2) densely covered by scales, completely covering integument, but not overlapping. Pronotal adpressed scales placed transversely with tips directed to midline, those on elytra pointing apicad. Elytral striae with very short, piliform setae. Antennal club densely tomentose. Tibiae without grooming patch. Ventral surface of body with sparse, adpressed to semierect piliform setae, integument clearly visible. Trochanteral setae present.

*Rostrum* in dorsal view (Fig. 3) short, transverse; epistome transverse, medially notched apically, more or less V-shaped, delimited behind by fine raised line, naked, shiny, with one row of long parepistomal setae on each side; frons undelimited; epifrons flat, at the same level as head, medially sulcate, epifrons and head with a dense longitudinal strigosity from anterior border of pronotum to epistomal margin, covered by scales, sides of epifrons concave, tapering apicad in basal half, subparallel in apical half, lateral margin moderately projecting, extended above eyes in a supraocular ridge. Antennal scrobes in dorsal view inconspicuous, visible only in short apical part as narrow furrows, pterygia weakly prominent, in lateral view (Fig. 4) deep, short, curved in front of eyes, not reaching lower margin of rostrum, with ventral edge slightly longer than dorsal one, separated from eye about half width of scrobe.

*Head* with eyes small, lateral, in dorsal view strongly convex, asymmetrical, highest point displaced backwards, in lateral view slightly oblong, separated from supraocular ridge by a fine furrow. Vertex wide, flat, without fovea. Mandibles trisetose, with round, flat scar. Prementum subtrapezoidal, with angles rounded, glabrous, asetose. Postmentum with 2 very long subapical setae. Almost adelognathous, prementum very narrowly separated from hypostoma.

*Antennae* (Fig. 8) very slender, 11-segmented. Scape reaching anterior border of pronotum when folded, slightly longer than funicle, bisinuate, at basal half extremely slender, in the apical half clavate. Desmomeres 7, first 2 elongate, last 4 moniliform. Club oval, slightly wider than apical part of scape.

**Figures 5–8.**
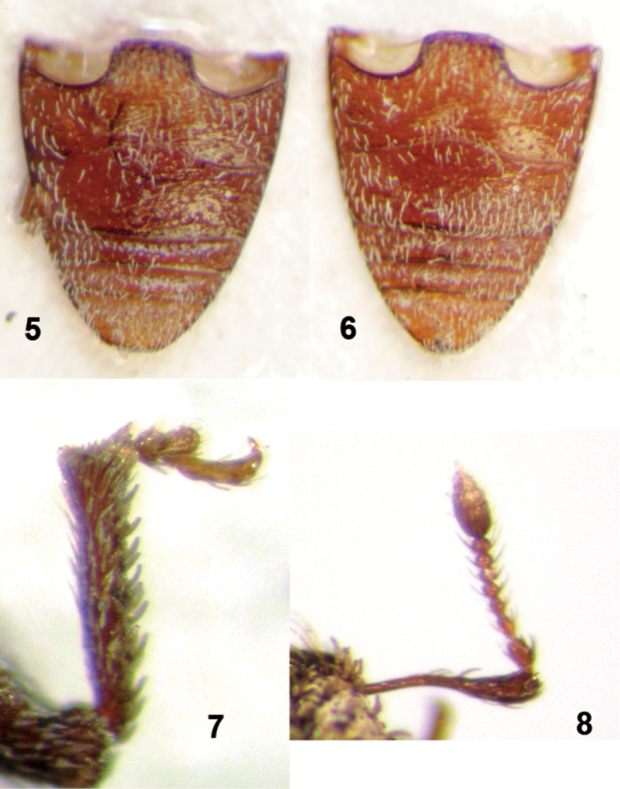
*Moreiba canariensis* (Franz): **5** Abdominal sternites, male **6** Abdominal sternites, female **7** Male right protibia, front view **8** Male right antenna.

*Pronotum* moderately wider than long, with rounded sides, anterior border distinctly narrower than posterior one, disc weakly curved, in the same curve as elytra, weakly depressed behind apical margin, without postocular lobes or setae, pronotal surface densely granulate. Base curved towards scutellum. Procoxae tangent, subglobular, situated at midlength of the pronotum.

*Scutellum* triangular, very small.

*Elytra* strongly coapted, not fused; in dorsal view subelliptical, base connivent with that of pronotum, with a narrow vertical step towards mesonotum except near scutellum, humeral calli absent; in side view weakly convex on dorsum, slope overhanging apex, with 10 complete, finely punctured striae, interstriae flat, ca. 3 × as wide as striae, these at apex join 1, 2, 3+8, 4+5, 6+7, 9, 10.

*Meso- and metaventrite*. Mesoventrite transversally depressed, in a more dorsal plane than metaventrite. Mesocoxae subglobular, mesoventral process narrow, about as wide as 1/5 of diameter of mesocoxa. Metaventrite between coxae about as long as mesocoxa. Metanepisternal suture complete, metanepisternum narrow, its base oblique, projecting over outer angle of metacoxae. Metacoxae oval, transverse, not visibly touching costal margin of elytra; abdominal process distinctly narrower than largest diameter of metacoxa, subtruncate. Metendosternite very short, with long furcal arms in a flat angle, hemiducts weakly developed, anterior tendons well separated. Metathoracic wings absent.

*Legs*. Femora of all legs edentate, medially swollen. Protibia (Fig. 7) in both sexes straight, apex slightly enlarged internally and externally, rounded, with a fringe of fine, yellow setae. Mucro of all legs not developed. Metatibial talus glabrous, slightly oblique, not ascending, without corbel or bevel. Tarsi slender, tarsomere 3 transverse, wider than the others, deeply bilobed, onychium strongly projecting beyond the lobes. Claws 2, equal, connate in basal half.

*Abdomen* (Figs 5–6). Abdominal ventrite 1 in midline a little longer than ventrite 2 (21:19); ventrite 2 clearly longer than ventrites 3 and 4 combined (19:11). Suture 1 fine, curved forward at middle; sutures 2, 3 and 4 straight, wide and deep. Fifth ventrite uniformly rounded in females, subtruncate at apex in males.

**Figures 9–14.**
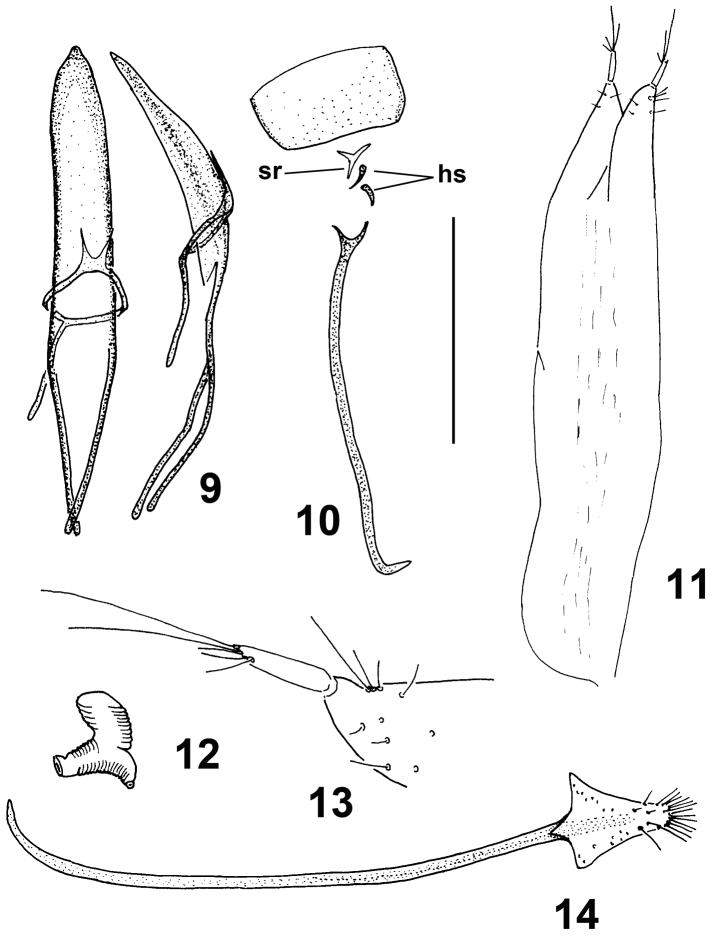
*Moreiba canariensis* (Franz): **9** Penis, dorsal and lateral **10** Male sternite VIII (*sr* spiculum relictum) and IX (*hs* hemisternites) **11** Female IX hemisternites and styli **12** Spermatheca **13** Detail of apex of female IX hemisternite and stylus **14** Female sternite VIII. Scale: **9–12, 14**: 0.5 mm; **13**: 0.125 mm.

*Male genitalia and terminalia*. Genitalia of the ‘pedal’ type (Alonso-Zarazaga 2007). Penis (Fig. 9) weakly sclerotised, slender, moderately long, pointed, temones slightly longer than pedon. Tegminal apodeme (manubrium) a little shorter than half the length of temones of penis; parameroid lobes long, narrow, fused at base, a little longer than half length of manubrium. Endophallus devoid of sclerotisations, with minute asperities. Sternite VIII membranous, *spiculum relictum* (Fig. 10) present, Y-shaped. Sternite IX (Fig. 10) with two small, comma-shaped sclerotized basal plates (hemisternites) and a long, narrow, Y-shaped *spiculum gastrale*, its apex turned 90°.

*Female genitalia and terminalia*. Sternite VIII (Fig. 14) with long and slender apodeme (*spiculum ventrale*), curved at apex, without differentiated caput and terminated about middle of lamella, this small, translucent, without margo basalis and apicalis, subtriangular, slender, with an apical tuft of setae. Hemisternites IX (gonocoxites) (Figs 11, 13) of ovipositor elongate, weakly sclerotised, with long slender apical styli with setae. Spermatheca (Fig. 12) C-shaped, with corpus and cornu inflated, visibly ringed, ramus and nodulus developed.

#### Note.

Sexual dimorphism not apparent, except for slight differences in fifth ventrite.

#### Distribution.

This genus is presently known from two islands of the Canaries: Gran Canaria and El Hierro, but some samples are known as well from Tenerife, La Palma, Fuerteventura, Lanzarote and Montaña Clara. These are in study and may represent different species.

#### Etymology.

Moreiba was the goddess of women and fertility among the ancient inhabitants of El Hierro (the ‘bimbaches’). Gender feminine.

### Moreiba canariensis

(Franz, 1995) comb. n.

http://species-id.net/wiki/Moreiba_canariensis

Strophosoma canariense Franz 1995: 37.

#### Description.

*Measurements* (in mm): Length: 2.55–2.75 (x– = 2.65, σ_n-1_ = 0.07, n= 8). Width: 1.40-1.48 (x– = 1.44, σ_n-1_ = 0.03, n = 8). Ranges given by Franz (1995) for the length are apparently taken from apex of rostrum, which is not standard in Curculionoidea, and width seems to be a gross underestimation.

*Integument* reddish brown to pitch brown.

*Body* densely covered by subtriangular scales, these apically awned (with a projecting bristle), adpressed to slightly raised. Head vestiture mostly creamy or whitish, that of pronotum different shades of brown, with one lateral band and a median thin band (sometimes incomplete anteriorly) creamy or whitish, elytra watery chequered in shades of brown, whitish and cream, 4^th^ interstria paler on declivity, 7^th^ and 8^th^ at base. Scutellum and legs with whitish scales. Semierect sublanceolate setae present among scales, on head forming a peculiar supraocular row, on pronotum denser, more perpendicular on sides, on elytra forming a regular row on interstriae. Semierect setae also present on antennae and legs.

*Rostrum* ca. 0.57 × as long as wide at base, as wide at pterygia as at base. Eyes asymmetrical, large-faceted, ommatidia separately convex, giving a blackberry aspect.

*Antennae* with scape ca. 7.0 × as long as wide, bisinuate, clubbed in apical half, desmomeres 1-3 oblong, pedicel about as long as desmomeres 2-3 together, desmomeres 4-7 moniliform. Club ca. 2.0 × as long as wide, oval, as long as the last 4 ½ desmomeres.

*Pronotum* ca. 0.80 × as long as wide. Surface under vestiture densely and irregularly granulate, granules flattened, small, ca. 30 µm in diameter.

*Elytra* ca. 1.30 × as long as wide, ca. 2.5 × as long as pronotum, interstrial setae longer on sides than on disc, apically rounded to subtruncate, with sides subparallel, on disc ca. half the width of an interstria, separated in the row by a distance about twice their length, interstrial adpressed scales in 4–5 irregular rows. Interstriae flat, smooth, minutely punctate. Striae with shortly subrectangular punctures separated a distance similar to their length, punctures bearing small, piliform scales not surpassing margins of puncture. Upper part of declivity slightly overhanging elytral apex.

*Legs* short, moderately robust, 1^st^ tarsomere oblong, 2^nd^ small, subtransverse, 3^rd^ strongly transverse, bilobed, onychium a little longer than 1^st^, surpassing lobes of 3^rd^ by 2/3 of its length.

*Wings*. Franz (1995) stated that the species was brachypterous. In the three specimens I have dissected into pieces, I have been unable to find any trace of a wing, the insects being totally apterous.

*Penis* in dorsal view with pedon ca. 4.5 × as long as wide, sides slightly converging towards ostium, apical plate subogival, apex slightly projecting, in side view slightly curved ventrally.

*Styli* of ovipositor ca. 4 × as long as wide.

*Spermatheca* with ramus curved opposite to cornu.

#### Material studied.

12 specimens, labelled El Hierro / Las Playas, ex coll. H. Franz, in coll. Alonso-Zarazaga (MNCN). The specimens have no date. They come from the type locality.

#### Biological notes.

Franz (1995) mentions the presence of the species in xerophytic habitats in the islands, mostly under *Periploca laevigata* Aiton (Apocynaceae). A. Machado (*pers. comm.*) reports specimens under the basal leaves of *Asphodelus* sp. (Xanthorrhoeaceae). This is probably another polyphagous genus.

## Discussion

The original placement of this species in the genus *Strophosoma* Billberg, 1820 is untenable, but easily understandable by the fact that Franz was not a specialist in Curculionoidea. In fact, the overall appearance and the size are more reminiscent of a *Trachyphloeus* Germar, 1817.

Of the characters stated by Machado (2010) to define the Laparocerini in the adult state, *Moreiba* matches each one, except the presence of mucronate tibiae. The character that Machado considered to be fundamental in the definition of the tribe (the presence of a *spiculum relictum* on the male VIII sternite) is present in *Moreiba*. Based on this definition, Machado (*l.c.*) excluded several genera from the tribe, and left the question open for three other genera: *Aphyonotus* Faust, 1895, *Asmaratrox* Heller, 1909 and *Straticus* Pascoe, 1886. The study of some specimens of *Asmaratrox intrusus* Heller, 1909 and of *Asmaratrox coxalis* Heller, 1909 in the collections of the NHM (London) and of the MNHN (Paris), and of specimens of *Teripelus brachyderoides* (Fairmaire, 1882), *Teripelus phoeostictus* (Fairmaire, 1882) and *Aseneciobius raffrayi* (Fairmaire, 1882) (Perrin and Alonso-Zarazaga 2012) has convinced me that they are closely related. Alonso-Zarazaga and Lyal (1999), following the current opinions, placed the genus *Asmaratrox* in Laparocerini, *Teripelus* Heller, 1909 in Omiini and *Aseneciobius* Hustache, 1939 in the Peritelini, reflecting thus the present chaos in Entiminae systematics. These three genera (and the Asian *Cyrtozemia* Pascoe, 1872) are related to *Systates* Gerstäcker, 1871 and belong to the so called ‘African’ Peritelini, characterized by the very short abdominal ventrite 2, about as long as the 3^rd^, and the straight suture 1. I have not studied *Straticus*, but it could also belong here. The placement of *Moreiba* in Laparocerini is also supported by molecular data (A. Machado, *pers. comm.*). As here restricted, Laparocerini includes only the genera *Laparocerus* and *Moreiba*, that can be separated by using the following key:

**Table:** 

1	Rostrum more or less subisodiametric. Head and rostrum punctate or punctulate, never strigose. Scape straight to moderately curved. Antennal club fusiform. Pronotum punctate or punctulate. Elytral declivity not overhanging apex. At least one pair of tibiae mucronate in males. Endophallus with visible spines, teeth or other sclerites. Length usually more than 3 mm	*Laparocerus*
–	Rostrum very transverse, less than 0.75× as long as wide. Head and rostrum with dense longitudinal strigosity. Scape bisinuate. Antennal club shortly oval. Pronotum granulate. Elytral declivity overhanging apex. All tibiae lacking mucro in both sexes. Endophallus devoid of visible sclerites. Length less than 3 mm	*Moreiba*

The presence of a *spiculum relictum* in Laparocerini is probably of the highest interest, as Machado (*l.c.*) has already pointed out, since it is absent in some Entiminae tribes (like Sciaphilini, Trachyphloeini, etc.). There is another peculiar feature that could be of some interest in characterizing the tribe Laparocerini, and that has been observed both in *Laparocerus* and in *Moreiba*. The basal margin of the metanepisternum protrudes obliquely over the outer angle of the metacoxa, hiding it in perpendicular view, so that the metacoxa does seem not to reach the elytral margin. In most Entiminae, the metanepisternal base ends transversely, not hiding the outer apex of the metacoxa, and the metacoxa is clearly seen touching the costal margin of the elytron. A survey of this character is needed to evaluate its phylogenetic significance.

The Laparocerini may be an evolutionary relict of former faunas that has been displaced by either climatological or competitive forces to a refuge in the Atlantic islands (Canaries and Madeira). There, at least *Laparocerus* has radiated into some two hundred species. The study of more *Moreiba* populations may help to cast light into the evolutionary history of this interesting taxon.

## Supplementary Material

XML Treatment for Moreiba

XML Treatment for Moreiba canariensis
